# Socioeconomic Predictors of Pituitary Surgery

**DOI:** 10.7759/cureus.3957

**Published:** 2019-01-25

**Authors:** Sayantan Deb, Daivik B Vyas, Arjun V Pendharkar, Paymon G Rezaii, Matthew K Schoen, Kaniksha Desai, Melanie H Gephart, Atman Desai

**Affiliations:** 1 Neurosurgery, Stanford University School of Medicine, Stanford, USA; 2 Internal Medicine, Stanford University School of Medicine, Stanford, USA

**Keywords:** pituitary, health determinants, pituitary adenoma, race, socioeconomic status, surgery

## Abstract

Background: There exists a lack of data on the effect of socioeconomic status (SES) on outcomes for pituitary tumors, which have been associated with significant morbidity. The goal of this population-level study is to investigate the role of SES on receiving treatment and survival in patients with pituitary tumors.

Methods: The Surveillance, Epidemiology, and End Results (SEER) program database from the National Cancer Institute was used to identify patients diagnosed with pituitary tumors between 2003 and 2012. SES was determined using a validated composite index. Race was categorized as Caucasian and non-Caucasian. Treatment received included surgery, radiation, and radiation with surgery. Odds of receiving surgery and survival probability were analyzed using multivariate logistic regression and Cox proportional hazards model, respectively.

Results: A total of 25,802 patients with pituitary tumors were identified for analysis. High SES tertile (odds ratio (OR) = 1.095; 95% confidence interval (CI) [1.059, 1.132]) and quintile (OR = 1.052; 95% CI [1.031, 1.072]) were associated with higher odds of receiving surgery (p<0.0001). Caucasian patients had higher odds of receiving surgery when compared to non-Caucasian patients (OR = 1.064; 95% CI [1.000, 1.133]; p<0.05). Neither SES nor race were significant predictors of survival probability.

Conclusion: Socioeconomic status and race were found to be associated with higher odds of receiving surgery for pituitary tumors, and thus serve as independent predictors of surgical management. Further studies are required to investigate possible causes for these findings.

## Introduction

Pituitary tumors are a heterogeneous collection of neoplasms that arise from the pituitary gland. They are the third most common intracranial tumor, with an estimated overall prevalence of 16.7% [1-2]. Surgical resection remains a mainstay treatment for functioning pituitary tumors and is indicated as first-line treatment in many instances, particularly when symptomatic mass-effect or visual deficits are present [3-5]. Studies on the efficacy of surgical resection suggest that surgery is able to successfully achieve hormonal resolution in upwards of 80% of tumor subtypes, with relatively low rates of complication and mortality [6]. Given the frequency of pituitary tumors, their associated morbidity, and the benefits from surgical intervention, there exists substantial interest in determining factors that influence early detection, management, and survival outcomes.

Socioeconomic status (SES) is a composite measure that includes variables pertaining to occupation, education, and income. SES has been found to be associated with disparities in incidence, treatment received (including radiation therapy, surgical resection, and adjuvant therapy), and survival outcomes for various cancers including lung, prostrate, breast, and cervix cancers [7-8]. SES also has also been associated with overall survival and odds of receiving treatment for intracranial tumors [9-10].

Race has also been found to be a predictor for variation in neuro-oncological care. In a study of 76,436 cases of tumor biopsy or resection, African Americans and Hispanics had disproportionately worse access to high-quality neuro-oncologic care over time compared with white patients [11]. Furthermore, a national study of over 84,000 patients showed that black patients were 38% less likely to receive surgery when compared to white patients, and black patients from high SES had lower surgical rates than white and Hispanic patients from low SES backgrounds [12]. Similar patterns have been found for lung, esophageal, and pancreatic cancers [13]. Limitations in access to quality care, as well as differences in patient engagement during provider selection, have previously been suggested to contribute to patterns of race-specific differences [14].

Currently, few studies have investigated racial and socioeconomic determinants of pituitary tumor outcomes. Prior analysis using the Surveillance, Epidemiology, and End Results (SEER) program database from at the National Cancer Institute has uncovered associations between pituitary adenoma incidence rates and age, gender, and race [15]. In another national study, SES and race were associated with differing odds of admittance to high-volume centers for pituitary adenoma patients undergoing trans-sphenoidal surgery [16]. In a single-center study, increased length of stay following trans-sphenoidal resection of pituitary adenoma was associated with lower SES [17]. These studies, however, utilized varying matrices of SES, employed single-center designs, and/or did not investigate rates of treatment received. Using a uniform and comprehensive index of SES developed by Yost et al., this national retrospective study aims to determine whether SES and race are determinants of surgical intervention and survival probabilities of patients with pituitary tumors [18].

## Materials and methods

Patients diagnosed with pituitary tumors between 2003 and 2012 were identified using a custom database consisting of 17 registries from the SEER program database of the National Cancer Institute. Study-eligible patients included those who had primary pituitary tumors, had a known age of diagnosis and were actively followed. The resulting cohort included approximately 28% of the patients in the United States with the diagnosis of pituitary tumor. Patient death information was gathered by the SEER program from the National Death Index, as well as state, local, and institutional records. Patient follow-up time was measured in months; patients were followed from the time of diagnosis (when they were reported to the SEER registry) to 60 months, until death, loss to follow up, or the study cutoff date (December 2012), whichever occurred first. Records from Alaska and Louisiana were excluded on the basis of incompleteness. All data from this database are de-identified, and thus this study is exempt from IRB approval in accordance with the Health Insurance Portability and Accountability Act of 1996.

A diagnosis of pituitary tumor was specified using the SEER variables “Histology record – Brain groupings” (for tumor type) and “Primary Site” (for tumor location). For the “Histology recode—brain groupings” variable, the value ‘Pituitary’ was used to specify tumor type. For the tumor site variable “Primary Site”, the value ‘C75.1 – Pituitary Gland’ was used. Additionally, the World Health Organization (WHO) grading criteria (Grades 1-4 and unknown), as adopted by the SEER classification system, was used to categorize patients according to tumor grade. SES was calculated using an index developed by Yost et al. [18]. The index scores patients based on national census-tract information at the time of entry into the registry, which includes SES markers such as education level, median income, median home value, median rent, poverty level, and employment status. The scores are then divided into tertiles (T1-T3) and quintiles (Q1-Q5). T1 and Q1 denote the lowest socioeconomic stratum, while T3 and Q5 denote the highest, and reflect the patient SES compared to a national population. Treatments received by patients were treated as binary variables, and included surgery, radiation, and radiation with surgery. These treatment categories were, however, not exclusive. Race was categorized as Caucasian or non-Caucasian based on self-reported data in the SEER database.

The odds of receiving treatment based on SES and race were determined using multivariable logistic regression. Survival probabilities, based on SES, race, and treatment modality, were analyzed using Cox proportional hazards model. All results were adjusted for age at diagnosis, sex, and tumor grade. P-values of less than 0.05 were considered significant. Data were analyzed using SAS v9.4 (SAS Institute Inc., Cary, NC, USA).

## Results

The study identified 25,802 patients diagnosed with pituitary tumors, with an average age at diagnosis of 50.18 years; 55.2% of participants in the sample were female, and 71.7% of patients in this cohort were Caucasian. When divided into SES tertiles, 32.6% of patients were in T1, and 19.2% patients were in Q1 when divided into quintiles. 45.2% of patients in this cohort received surgery, 4.7% received radiation, and 3.7% received radiation combined with surgery. Median follow-up time was 42 months (Table 1).

**Table 1:** Study Population Demographics N = number of participants; SEM = standard error of the mean.

Study Population Demographics	
N	25,802
Sex, N [%]	
Male	11,550 [44.8]
Female	14,252 [55.2]
Race, N [%]	
Caucasian	18,508 [71.7]
Non-Caucasian	6,818 [26.4]
Unknown	476 [1.85]
Mean age at diagnosis ± SEM, years	50.18 ± 0.11
Tertile distribution, N [%]	
Tertile 1	8,410 [32.6]
Tertile 2	8,643 [33.5]
Tertile 3	8,749 [33.9]
Quintile distribution, N [%]	
Quintile 1	4,945 [19.2]
Quintile 2	5,196 [20.1]
Quintile 3	5,197 [20.1]
Quintile 4	5,168 [20.0]
Quintile 5	5,296 [20.5]
Treatment, N [%]	
Surgery	9,872 [45.2]
Radiation	1,210 [4.73]
Radiation with Surgery	973 [3.77]
Median Follow-up, Months	42
Dead at Study Cutoff, N [%]	9 [0.03]
Lost to Follow-up, N [%]	17,257 [66.9]

Effect of SES on treatment received

After adjusting for age at diagnosis, sex, race, and tumor grade, high SES tertile (odds ratio (OR)=1.095) and quintile (OR=1.052) were found to be associated with higher odds of receiving surgery (p<0.0001) (Table 2). There were no significant associations between SES and receiving radiation or radiation with surgery. The distributions of patients who received surgery, radiation, or radiation with surgery across SES strata are summarized in Tables 3A-3B for SES tertiles and quintiles, respectively.

**Table 2:** Effect of High SES on Treatment Received OR = odds ratio; SES = socioeconomic status.

	OR^a^	95% Confidence Interval	p-value
Treatment for SES Tertiles			
Surgery	1.095	(1.059 – 1.132)	<0.0001
Radiation	0.948	(0.883 – 1.019)	0.1493
Radiation with Surgery	0.968	(0.894 – 1.048)	0.4214
Treatment for SES Quintiles			
Surgery	1.052	(1.031 – 1.072)	<0.0001
Radiation	0.962	(0.923 – 1.003)	0.0719
Radiation with Surgery	0.975	(0.931 – 1.021)	0.2746
Reference = Lowest SES (lowest 3rd tertile or 5th quintile, respectively)			
^a ^Adjusted for Age at Diagnosis, Sex, Race, and Tumor Grade			

**Table 3:** Treatment Received by SES Tertile (A) and Quintile (B) N = number of participants; SES = socioeconomic status. T1-T3 are 'Tertiles' and 'Quintiles' are Q1-Q5.

A. Treatment Received (N [%]) by SES Tertile			
	T1	T2	T3
Surgery	3,068 [31.1]	3,286 [33.3]	3,518 [35.6]
Radiation	409 [33.8]	403 [33.3]	398 [32.9]
Radiation with Surgery	323 [33.2]	325 [33.4]	325 [33.4]

B. Treatment Received (N [%]) by SES Quintile
	Q1	Q2	Q3	Q4	Q5
Surgery	1,812 [18.4]	1,934 [19.6]	1,976 [20.0]	1,954 [19.8]	2,196 [22.2]
Radiation	232 [19.2]	268 [22.2]	247 [20.4]	224 [18.5]	239 [19.8]
Radiation with Surgery	180 [18.5]	217 [22.3]	197 [20.3]	186 [19.1]	193 [19.8]

Effect of race on treatment received

After adjusting for age at diagnosis, sex, socioeconomic status, and tumor grade, Caucasian patients were found to have higher odds of receiving surgery when compared to non-Caucasian patients (OR=1.064, p<0.05). Race was not a significant predictor of receiving radiation therapy or radiation with surgery (Table 4). The distribution of treatment received stratified by race is summarized in Table 5.

**Table 4:** Effect of Caucasian Race on Treatment Received OR = odds ratio; SES = socioeconomic status.

	OR^a^	95% Confidence Interval	p-value
Surgery	1.064	(1.000 – 1.133)	0.0488
Radiation	1.125	(0.982 – 1.287)	0.0886
Radiation with Surgery	1.107	(0.953 – 1.285)	0.1846
Reference = Non-Caucasian race			
^a^ Adjusted for Age at Diagnosis, Sex, SES, and Tumor Grade			

**Table 5:** Treatment Received by Race N = number of participants.

	Caucasian, N [%]	Non-Caucasian, N [%]
Surgery	7,224 [74.2]	2,511 [25.8]
Radiation	900 [74.9]	301 [25.1]
Radiation with Surgery	725 [74.9]	243 [25.1]

Effect of SES and race on survival probability

There was no significant effect of race on survival probability when adjusted for age at diagnosis, sex, SES, and tumor grade (hazard ratio (HR)=3.014, p>0.05). Neither high SES tertile (HR=0.786) nor quintile (HR=0.799) were significant predictors of survival probability when adjusted for age at diagnosis, sex, race, and tumor grade (p>0.05). Furthermore, neither surgery (HR=0.161) nor radiation with surgery (HR=0.358) were significant predictors of survival when adjusted for age of diagnosis, sex, race, and tumor grade (p>0.05).

## Discussion

This national study of 25,802 patients diagnosed with pituitary tumors investigated the role of SES and race on treatment received and overall survival. Our analysis found associations between high SES tertile/quintile and increased likelihood of receiving surgery, after adjusting for age at diagnosis, sex, race, and tumor grade. This finding is consistent with associations found in previous analyses which examined pituitary disease procedures at high-volume centers [16]. Given the multitude of factors that influence socioeconomic status, various elements may contribute to disparities seen in receiving treatment. Access to surgery may be influenced in part by financial determinants such as household income, insurance status, and regional coverage, which have been shown to impact access to surgical treatment for various cancer types [19-21]. Moreover, geographic characteristics, such as hospital volume and local referral patterns, may influence treatment selection by influencing both physical access to care as well as the quality of care. Further studies are required to explore the role of specific SES factors that impact treatment.

Caucasian race was also found to be associated with higher odds of receiving surgery, after adjusting for age at diagnosis, sex, SES, and tumor grade. Results from several nationwide studies confirm this finding, having demonstrated that the Caucasian race is an independent predictor for receiving surgical treatment for pituitary tumors [13,16,20,22]. These findings may be the result of multiple factors. First, there may be an inherent difference in pituitary tumor incidence across races. A previous analysis by McDowell et al. using the SEER database demonstrated that incidence rates of pituitary tumors varied according to age and race [15]. They found that age-adjusted incidence rates from 2004-2007 were significantly higher for African-Americans (4.4 per 100,000) compared to American Indians/Alaskan Natives (1.9 per 100,000), Asians/Pacific Islanders (2.3 per 100,000), and Caucasians (2.5 per 100,000) [15].

The possibility that Caucasian patients are predisposed to particular subtypes of pituitary tumors may also explain their increased likelihood of requiring and thus receiving surgery, for example, for non-prolactinomas, or more rapidly-growing tumors that cause visual or endocrinological abnormalities [15]. There has been relatively little investigation thus far on the extent to which pituitary hormonal characteristics vary according to geography and race; however, a study by Cimpean et al. found significantly different immunohistochemical and hormonal profiles in patients with pituitary adenomas in two separate European countries [23]. Their results may lend support to the concept of specific epidemiological factors affecting the biological characteristics of pituitary tumors.

Another possible explanation for the effect of race on treatment stems from evidence demonstrating the disproportionality in pituitary tumor diagnosis as incidental findings across patient populations [15]. When diagnosed as incidental findings, tumors may be clinically insignificant and therefore, not require surgical management. For example, African American patients, who experience higher rates of stroke compared to Caucasians, would necessarily undergo higher rates of brain imaging, and consequently may have higher rates of incidental diagnoses of pituitary tumors on imaging. Thus, due to these inherent differences in imaging rates, non-white patients may be more likely to have asymptomatic pituitary tumors found incidentally, and less likely to undergo surgery [15].

Finally, these findings may be also explained by the differences in healthcare accessibility for various ethnicities. Prior analysis of national Medicare data found that African-American patients are overwhelmingly seen at a smaller concentration of hospitals, suggesting racial differences in access to care [24]. Furthermore, Liu et al. reported in an analysis of surgical procedures across hospitals in California that non-white patients have a significantly reduced likelihood of being seen at high volume hospitals relative to white patients [25]. Thus, the relatively limited access to healthcare for non-white patients may underlie the racial variances observed in this study. It is also possible that our findings were influenced by additional systemic biases previously observed for physician decision-making and management between white and non-white patients [26].

Surgery remains a common treatment for pituitary tumors, and inability to receive surgery can contribute significantly to morbidity. Surgery is typically indicated for visual field loss, excess hormonal production, and tumor progression in pituitary tumors other than prolactinomas. For prolactinomas, dopamine agonists are commonly used as adjuvant therapy; however, surgery remains an effective treatment option in certain clinical scenarios including visual field loss, and intolerance or resistance to dopamine agonists [27]. The pattern of treatment modality utilization found in this study is similar to those observed in other national-level reports. Olsson et al. found, in a cohort of 2,795 non-functional pituitary adenoma patients, a 53% rate of surgery alone [28]. Similarly, Lesén et al. demonstrated a frequency of 60% for surgery as the first-line treatment in a Sweden-wide cohort of 358 acromegaly patients [29]. Consequently, socioeconomic and racial effects of receiving surgical treatment hold significant implications for the quality of life of patients with pituitary tumors. In the absence of surgical treatment, symptomatic patients may continue to experience the debilitating conditions that result from these tumors, such as Cushing’s disease and acromegaly, or loss of peripheral vision that results from mass effects. However, the significant morbidity associated with these conditions is not reflected in the outcome of overall survival as measured in this study.

This study found no association between SES or race and receiving radiation or radiation with surgery. In addition, no significant racial, socioeconomic, or treatment-specific associations with survival probability in pituitary tumors were found, likely due to the relatively low mortality of these tumors. In the case of surgically-managed pituitary tumors, mortality across all subtypes ranges from 1%-8%, with an overall mortality of 1.6% [30]. Detection of subtle differences in mortality may thus require a significantly longer time period than the five-year window used in this study. Future studies assessing specific symptoms (e.g., visual loss), disability, hormonal cure, and other quality of life metrics may allow further insight into outcome variation.

Limitations of this study include restrictions associated with treatment and outcome variables of the database, as well as lack of subcategory tumor-type classifications. The SEER database does not include information regarding medical management of pituitary tumors and the use of hormonal replacement; nor does it contain information about the use of chemotherapy or dopamine agonists. The database also does not parse out subcategories and histological variants of pituitary masses. This study also does not stratify non-Caucasian patients into different ethnicities, as it was designed to detect significant differences between two sufficiently powered patient cohorts. There may be, however, further heterogeneity in treatment receipt based on race, which may be investigated in a future study with a larger number of non-Caucasian patients.

Future work which examines the role of specific SES factors (e.g., financial and geographic determinants) and subcategories of race may further elucidate the causes of trends identified in this study. In addition, prospective studies designed to further stratify patients based on tumor genetics and histopathology prior to outcomes analysis may uncover factors that explain disparities in access and quality of care for pituitary tumors. Ongoing public health research within this domain can help answer questions that may lead to the development of targeted interventions for improved access to pituitary cancer treatment.

## Conclusions

In conclusion, this national study of 25,802 patients identifies socioeconomic status and race as significant predictors of receiving surgery for pituitary tumors. These findings contribute to the growing body of literature analyzing demographic influences on cancer outcome. Further investigation is required to understand possible reasons for these associations, to help ensure optimal care across the entire population.

## References

[1] Dolecek TA, Propp JM, Stroup NE, Kruchko C (2012). CBTRUS statistical report: primary brain and central nervous system tumors diagnosed in the United States in 2005-2009. Neuro Oncol.

[2] Ezzat S, Asa SL, Couldwell WT, Barr CE, Dodge WE, Vance ML, McCutcheon IE (2004). The prevalence of pituitary adenomas: a systematic review. Cancer.

[3] Freda PU, Beckers AM, Katznelson L, Molitch ME, Montori VM, Post KD, Lee Vance M (2011). Pituitary incidentaloma: an endocrine society clinical practice guideline. J Clin Endocrinol Metab.

[4] Sivakumar W, Chamoun R, Nguyen V, Couldwell WT (2011). Incidental pituitary adenomas. Neurosurg Focus.

[5] Villwock JA, Villwock M, Deshaies E, Goyal P (2014). Significant increases of pituitary tumors and resections from 1993 to 2011. Int Forum Allergy Rhinol.

[6] Tabaee A, Anand VK, Barrón Y (2009). Endoscopic pituitary surgery: a systematic review and meta-analysis. J Neurosurg.

[7] Clegg LX, Reichman ME, Miller BA (2009). Impact of socioeconomic status on cancer incidence and stage and end results: national longitudinal mortality study. Cancer Causes Control.

[8] Albano JD, Ward E, Jemal A (2007). Cancer mortality in the United States by education level and race. J Natl Cancer Inst.

[9] Deb S, Pendharkar AV, Schoen MK, Altekruse S, Ratliff J, Desai A (2017). The effect of socioeconomic status on gross total resection, radiation therapy and overall survival in patients with gliomas. J Neurooncol.

[10] Inskip PD, Tarone RE, Hatch EE (2003). Sociodemographic indicators and risk of brain tumours. Int J Epidemiol.

[11] Mukherjee D, Zaidi HA, Kosztowski T, Chaichana KL, Brem H, Chang DC (2010). Disparities in access to neuro-oncologic care in the United States. Arch Surg.

[12] Liederbach E, Lewis CM, Yao K, Brockstein BE, Wang CH, Lutfi W, Bhayani MK (2015). A contemporary analysis of surgical trends in the treatment of squamous cell carcinoma of the oropharynx from 1998 to 2012: a report from the national cancer database. Ann Surg Oncol.

[13] Al-Refaie WB, Muluneh B, Zhong W, Parsons HM, Tuttle TM, Vickers SM, Habermann EB (2012). Who receives their complex cancer surgery at low-volume hospitals?. J Am Coll Surgeons.

[14] Freedman RA, Kouri EM, West DW, Keating NL (2015). Racial/ethnic differences in patients’ selection of surgeons and hospitals for breast cancer surgery. JAMA Oncol.

[15] McDowell BD, Wallace RB, Carnahan RM, Chrischilles EA, Lynch CF, Schlechte JA (2011). Demographic differences in incidence for pituitary adenoma. Pituitary.

[16] Mukherjee D, Zaidi HA, Kosztowski T, Chaichana KL, Salvatori R, Chang DC, Quiñones-Hinojosa A (2009). Predictors of access to pituitary tumor resection in the United States, 1988-2005. Eur J Endocrinol.

[17] Sarkiss CA, Lee J, Papin JA (2015). Pilot study on early postoperative discharge in pituitary adenoma patients: effect of socioeconomic factors and benefit of specialized pituitary centers. J Neurol Surg B Skull Base.

[18] Yost K, Perkins C, Cohen R, Morris C, Wright W (2001). Socioeconomic status and breast cancer incidence in California for different race/ethnic groups. Cancer Causes Control.

[19] Seyedin S, Luu C, Stabile BE, Lee B (2012). Effect of socioeconomic status on surgery for pancreatic adenocarcinoma. Am Surg.

[20] Shapiro M, Chen Q, Huang Q (2016). Associations of socioeconomic variables with resection, stage, and survival in patients with early-stage pancreatic cancer. JAMA Surg.

[21] Smith JK, Ng SC, Zhou Z, Carroll JE, McDade TP, Shah SA, Tseng JF (2013). Does increasing insurance improve outcomes for US cancer patients?. J Surg Res.

[22] Weng Y, Korte JE (2012). Racial disparities in being recommended to surgery for oral and oropharyngeal cancer in the United States. Community Dent Oral Epidemiol.

[23] Cimpean AM, Melnic E, Bălinişteanu B, Corlan A, Coculescu M, Rusu S, Raica M (2015). Geographic-related differences of pituitary adenomas hormone profile: analysis of two groups coming from southeastern and eastern Europe. Int J Endocrinol.

[24] Jha AK, Orav EJ, Li Z, Epstein AM (2007). Concentration and quality of hospitals that care for elderly black patients. Arch Intern Med.

[25] Liu JH, Zingmond DS, McGory ML, SooHoo NF, Ettner SL, Brook RH, Ko CY (2006). Disparities in the utilization of high-volume hospitals for complex surgery. JAMA.

[26] Levinson W, Kao A, Kuby A, Thisted RA (2005). Not all patients want to participate in decision making: a national study of public preferences. J Gen Intern Med.

[27] Molitch ME (2017). Diagnosis and treatment of pituitary adenomas: a review. JAMA.

[28] Olsson DS, Bryngelsson IL, Ragnarsson O (2017). Time trends of mortality in patients with non-functioning pituitary adenoma: a Swedish nationwide study. Pituitary.

[29] Lesén E, Granfeldt D, Houchard A (2017). Comorbidities, treatment patterns and cost-of-illness of acromegaly in Sweden: a register-linkage population-based study. Eur J Endocrinol.

[30] Loyo-Varela M, Herrada-Pineda T, Revilla-Pacheco F, Manrique-Guzman S (2013). Pituitary tumor surgery: review of 3004 cases. World Neurosurg.

